# Store-directed price promotions and communications strategies improve healthier food supply and demand: impact results from a randomized controlled, Baltimore City store-intervention trial

**DOI:** 10.1017/S1368980017000064

**Published:** 2017-02-22

**Authors:** Nadine Budd, Jayne K Jeffries, Jessica Jones-Smith, Anna Kharmats, Ann Yelmokas McDermott, Joel Gittelsohn

**Affiliations:** 1 Division of Nutrition, Physical Activity, and Obesity, Centers for Disease Control and Prevention, 4770 Buford Highway, Mailstop F-77, Atlanta, GA 30341, USA; 2 The Gillings School of Global Public Health, University of North Carolina at Chapel Hill, Chapel Hill, NC, USA; 3 Department of Health Services & Nutrition Sciences Program, University of Washington, Seattle, WA, USA; 4 The Global Obesity Prevention Center, Bloomberg School of Public Health, Johns Hopkins University, Baltimore, MD, USA

**Keywords:** Obesity, Food stores, Trade promotions, Food access interventions, Pricing interventions

## Abstract

**Objective:**

Small food store interventions show promise to increase healthy food access in under-resourced areas. However, none have tested the impact of price discounts on healthy food supply and demand. We tested the impact of store-directed price discounts and communications strategies, separately and combined, on the stocking, sales and prices of healthier foods and on storeowner psychosocial factors.

**Design:**

Factorial design randomized controlled trial.

**Setting:**

Twenty-four corner stores in low-income neighbourhoods of Baltimore City, MD, USA.

**Subjects:**

Stores were randomized to pricing intervention, communications intervention, combined pricing and communications intervention, or control. Stores that received the pricing intervention were given a 10–30% price discount by wholesalers on selected healthier food items during the 6-month trial. Communications stores received visual and interactive materials to promote healthy items, including signage, taste tests and refrigerators.

**Results:**

All interventions showed significantly increased stock of promoted foods *υ*. control. There was a significant treatment effect for daily unit sales of healthy snacks (*β* = 6·4, 95% CI 0·9, 11·9) and prices of healthy staple foods (*β* = −0·49, 95% CI −0·90, −0·03) for the combined group *υ*. control, but not for other intervention groups. There were no significant intervention effects on storeowner psychosocial factors.

**Conclusions:**

All interventions led to increased stock of healthier foods. The combined intervention was effective in increasing sales of healthier snacks, even though discounts on snacks were not passed to the consumer. Experimental research in small stores is needed to understand the mechanisms by which store-directed price promotions can increase healthy food supply and demand.

Obesity is a profound problem both domestically and worldwide, causing those afflicted to lead shorter and less healthy lives and costing the USA an estimated $US 147 billion per year in direct health-care costs^(1)^. Public health experts recognize that changes in the food system over the last 40 years are a major driver of the obesity epidemic and that reversal or prevention of the epidemic is unlikely without improvements at multiple levels of the food environment^(2)^. In the USA, populations with low socio-economic status are disproportionately burdened by obesity and diet-related diseases, partially due to limited food resources within surrounding neighbourhoods^(3–5)^. Public health interventions that have sought to improve healthy food availability and access in small food stores located in low-income areas have seen moderate success; however, there is little to no research on the effects of price manipulations on consumer food behaviours in these settings, which operate with higher food costs and smaller economies of scale, and whose patrons are likely more price-sensitive^(6,7)^. Taxes on unhealthy food items hold promise, but are opposed by the food industry^(7,8)^. Conversely, subsidization of fruits and vegetables to improve availability and consumption is effective, but costly, and may not create total energy deficits if consumption of energy-dense foods via substitution effects is not simultaneously reduced^(9–11)^.

Employing industry-driven trade promotions is an alternative approach and has not been tested as an obesity prevention strategy. ‘Trade promotions’ are incentives (financial or otherwise) offered by manufacturers to retailers, rather than directly to consumers (i.e. ‘consumer promotions’)^(12)^. They are ubiquitous in supermarkets and are used to increase brand loyalty and boost sales of certain products during specific periods of time^(13–15)^. A performance-based allowance (PBA) is a type of trade promotion whereby a financial incentive is directed to the retailer in return for performing an activity requested by the supplier^(12,16)^. Retailers benefit from PBA in two ways: either by buying at discounted prices and selling at normal prices, or by increasing sales volume when they pass on some of the saving to customers (‘retail pass-through’). A food supplier (wholesalers who sell to and supply retailers directly and indirectly, e.g. manufacturer, vendor, broker, reseller) may offer price discounts on future cases of product if the retailer reaches a certain sales minimum (also called ‘movement allowance’), or a beverage supplier may pay an introductory allowance for products to be placed in the front of the store (also called ‘slotting allowance’). Food suppliers could support increasing healthy food purchases by utilizing PBA to shift consumer food preferences towards their ‘better-for-you’ or lower-calorie product lines. These products may not be considered ‘healthy’ by some nutrition experts, but they can provide the energy reduction needed for long-term weight loss and may also help ‘retrain’ consumers’ taste preferences towards healthier products^(17)^. Additionally, industry-led initiatives to reduce energy through portion size reductions, reformulation and marketing have resulted in superior sales and profit growth^(18)^. This approach would provide the food industry a mechanism by which to contribute to a healthier food supply without government intervention, while supporting corporate bottom lines.

The B’More Healthy Retail Rewards (BHRR) intervention trial sought to increase the availability and sales of select healthy foods in Baltimore’s small food stores by testing PBA and promotional strategies. PBA are underutilized in Baltimore’s small urban food stores (N Budd, unpublished results) to increase food sales, but are used heavily by the tobacco industry^(19)^. To our knowledge, the present study is the first store-based intervention trial to incorporate local food wholesalers and the first trial to test the effect of trade promotions on healthy food supply and demand in small stores. We examined the effects of performance-based monetary incentives (10–30% wholesale discount) and communications strategies, separately and combined, on small storeowners’ self-reported stocking, self-reported sales and prices of promoted healthier foods, and on related storeowner psychosocial variables. Our study’s hypothesis was that intervention stores (owners) would demonstrate significantly greater change in promoted food stocking, sales and psychosocial factor scores as compared with control stores from baseline to post-intervention, and that combined intervention stores would see the greatest change as compared with single intervention stores and controls. Our secondary research question assessed whether storeowners in the pricing intervention complied with the agreements of the PBA (stocking the item and retail pass-through).

## Methods

### Study setting and design

**Fig. 1:**
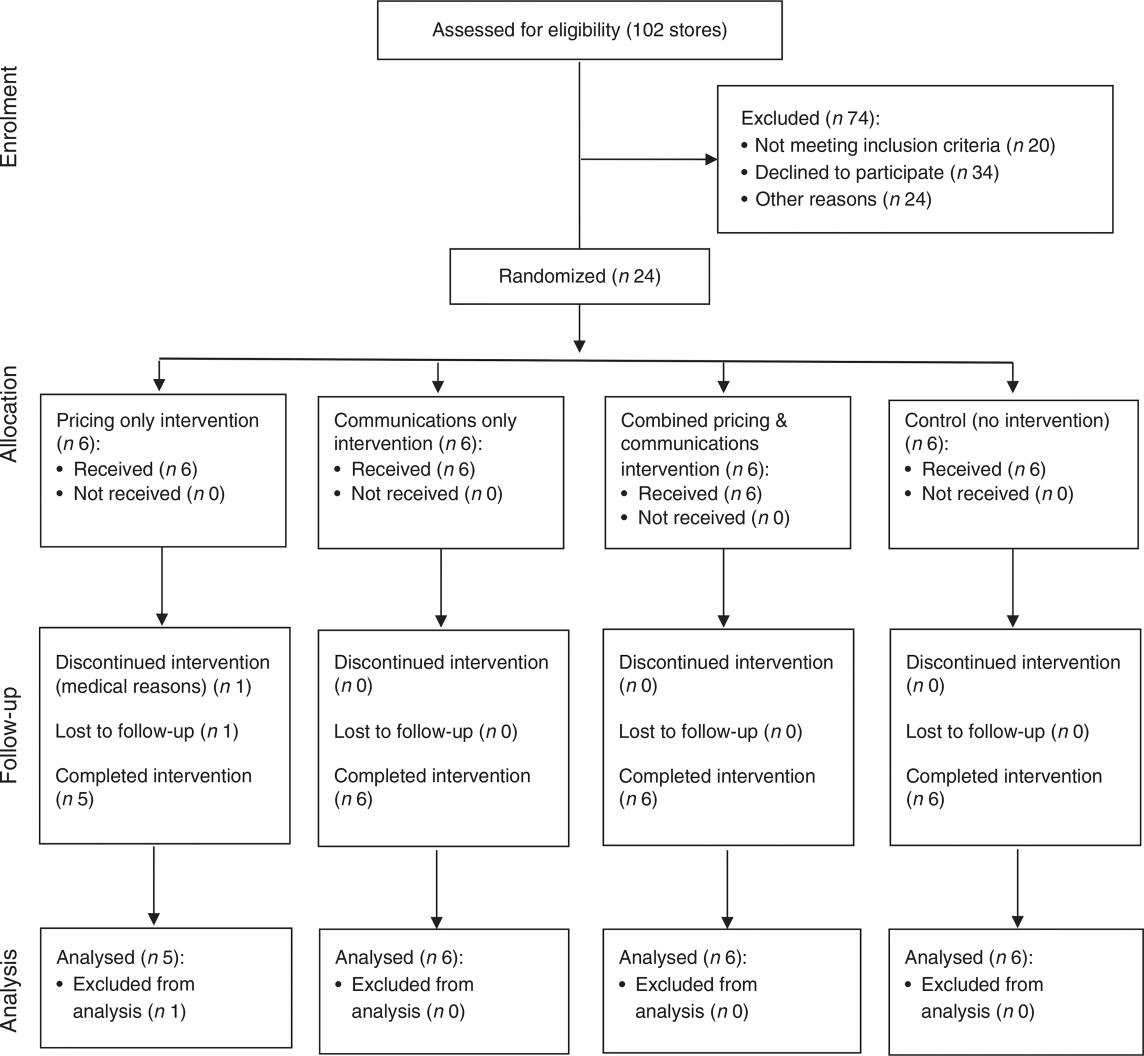
CONSORT (Consolidated Standards of Reporting Trials) flow diagram

BHRR was a 2 × 2 factorial randomized controlled trial (1:1:1:1) conducted from February to August 2013 in twenty-four small food stores and two food wholesale stores in Baltimore City, USA. Baltimore is the largest city in the state of Maryland with 621 849 residents, of whom 64% are African American and 24% live below the poverty line^(20)^. Store recruitment occurred from October to November 2012. Participant recruitment and flow through the study are presented in Fig. 1. Eligible corner stores were located in a low-income census tract (>50% living below the poverty level) at least 0·40 km (¼ mile) from each other and where greater than 75% of residents self-identified as African American.

After baseline data collection, stores were randomly allocated to one of four treatment groups: pricing only (G1; *n* 6), communications only (G2; *n* 6), combined pricing and communications (G3; *n* 6) or control (G4; *n* 6). To ensure comparison of groups with similar characteristics, store groups were stratified by two levels: participation in the Special Supplementation Nutrition Program for Women, Infants, and Children (WIC) and daily sales volume. Sales volume was used as a proxy for daily sales revenue, since storeowners were reluctant to share revenue estimates with research staff. Similarly, WIC status was used as a proxy for healthy food stocking, since stores carrying WIC must have a minimum required stock of healthy foods at all times. Thus, stratification occurred four ways: (i) high sales volume stores with WIC; (ii) high sales volume stores without WIC; (iii) low sales volume stores with WIC; and (iv) low sales volume stores without WIC. Store randomization occurred in a Baltimore City recreation centre where volunteers from the community drew store names from a bowl for one stratified group at a time (i.e. high sales volume WIC stores, etc.), so that the first drawing was assigned to G1 (pricing only), the second drawing was assigned to G2 (communications only), the third drawing was assigned to G3 (combined) and the fourth drawing was assigned G4 (control). This step was repeated with each stratified group until all stores were assigned a treatment group. Neither study participants nor research staff were blinded to the treatment arms due to the nature of the intervention design.

Sample size was determined *a priori* based on the parent study’s hypotheses on consumer-level outcomes^(21)^. For the present study, power analysis was conducted on a sample of twenty-four stores, with the primary outcome being the percentage change in sales between two groups. Outcomes from an earlier Baltimore-based store-intervention trial were used to calculate values for (i) mean change in sales between treatment groups and (ii) the sd^(22)^. A sample size of 6 in each group ensured 80% power to detect a difference in means of 2·4 (e.g. the difference between unit sales of G1 mean (*μ*_1_) of 4 bottles of water *υ*. a G2 mean (*μ*_2_) of 6 bottles of water) assuming that the common sd was 3·29 using a two-group *t* test with a 0·025 two-sided significance level.

### Intervention strategies

The BHRR intervention was conducted from February to August 2013 in twenty-four corner stores and two wholesale stores in Baltimore City. BHRR worked directly with one wholesaler at both of its locations on east and west sides of the city. The 6-month intervention was divided into three 8- to 10-week phases: (i) Better Beverages; (ii) Healthier Essentials (Staple Foods); and (iii) Healthier Snacks^(21)^. Each phase built upon the previous so that by the third phase, all foods and beverages were promoted simultaneously. Pricing intervention stores (G1 and G3) were given a 10–30% price discount on selected healthier food items, such as reduced-calorie sodas, frozen vegetables and whole-wheat bread, at the point of purchase from two food wholesale stores during the 6-month trial. Storeowners receiving the wholesale pricing discounts (i.e. those in G1 (pricing alone) and G3 (combination price and communications)) were asked to (i) stock the item and (ii) to pass partial or full discounts to customers (retail pass-through). BHRR grant funding was used to offset reduced costs of the selected foods at the wholesale stores.

Communications stores (G2 and G3) received visual, structural and interactive materials to promote healthy items, including signage, taste tests and small produce refrigerators. Additionally, communications stores also received laminated lists of promoted foods by phase that included information on their locations and prices at the wholesaler, and added suggestions on how to promote the foods in their stores using BHRR materials (i.e. shelf talkers, bags, etc.). At both wholesale stores, BHRR logo stickers were affixed on the shelves above or adjacent to the promoted products, so that intervention storeowners could easily recognize them.

A detailed description of BHRR’s study design is given elsewhere^(21)^. Intervention phases and treatment arms are outlined in Table 1.

### Data collection

The Store-impact Questionnaire (SIQ) was administered to storeowners once at baseline (December 2012–January 2013) and again at post-intervention (November 2013–January 2014). It gathered information on store (owner) demographic factors, sales and stocking of fifteen promoted foods, price of promoted foods, storeowner psychosocial factors including self-efficacy and intentions to stock, promote and sell promoted beverages/foods, and outcome expectations related to promoted food sales and to overall programme impact. The SIQ collected sales, stocking and pricing data on the following promoted items: Deer Park water, Pepsi Next, Coke Zero, Rutter’s 1% milk, Old Tyme 100% Whole Wheat Bread, Chunk Light tuna in water, Albacore tuna in water, Hanover or Bird’s Eye mixed frozen vegetables, Hanover or Bird’s Eye green frozen vegetables, Hanover or Bird’s Eye starchy frozen vegetables, apples, oranges, bananas, Quaker Oats low-fat granola bars and Utz baked potato chips. The SIQ is a pre-tested, standardized instrument that has been used previously in Baltimore stores^(21–23)^.

Interviews with storeowners were conducted in stores by the authors and other members of the research staff. Interviews with Korean-speaking owners were conducted in Korean and translated to English by Korean-speaking research staff. English versions of forms were used for all data collection.

### Data analysis

#### Dependent variables

All outcomes of interest were treated as continuous variables and include store stocking, sales and price changes of promoted food items, and related storeowner psychosocial factors. Average daily unit sales were assessed with fifteen questions (i.e. ‘How many units of Utz baked potato chips were sold per day in the last 30 d?’). Units were summed to create an average daily total. Stocking was assessed with fifteen questions (i.e. ‘Were Utz baked potato chips in stock in the last 30 d?’) and verified visually by data collectors. One point was given for each of the fifteen foods stocked in the last 30 d. For example, a store that stocked frozen broccoli, Coke Zero, bottled water and fresh apples obtained a total stocking score of 4. Points were summed to create a stocking score (possible range 0–15).

Prices of promoted foods that were stocked at both baseline and post-intervention collections were summed to create total food prices for each phase. If a food was not stocked at both collections, a 0 was imputed for both collections so that total change in price from baseline was 0. If a food was not stocked for one collection but was for another, the same price was imputed for both collections so that total change in price from baseline was 0.

Each of the psychosocial constructs (i.e. self-efficacy to stock, intentions to stock, outcome expectations for sales, outcome expectations for overall programme impact) were assessed with fifteen questions, each using a 5-point Likert scale that included ‘strongly agree’ (2), ‘agree’ (1), ‘undecided’ (0), ‘disagree’ (−1) and ‘strongly disagree’ (−2). Responses were summed to create the scale score for each category, each with a scale range of −30 to 30 points. All scales were tested for reliability using Cronbach’s *α*. An *α* of ≥0·70 was used to confirm good internal consistency and reliability^(24)^. For the fifteen questions evaluating self-efficacy for stocking promoted foods (i.e. ‘I can stock 100% whole-wheat bread in my store’), mean baseline score = 10·0 (sd 8·2); *α* = 0·84. For the fifteen questions evaluating intentions to sustain stocking of promoted foods (i.e. ‘I will stock frozen vegetables in my store after the programme is completed’), mean baseline score = 12·5 (sd 8·7); *α* = 0·87. For outcome expectations for promoted food sales assessed with fifteen questions (i.e. ‘Baked potato chips will sell well in my store’), mean baseline score = 6·1 (sd 7·3); *α* = 0·73. For outcome expectations on overall programme impact assessed with fifteen questions (i.e. ‘If I receive a produce refrigerator for my store, fresh fruit/vegetable sales will increase’), mean baseline score = 10·0 (sd 8·8); *α* = 0·93.

#### Baseline differences

Demographic measures included gender, self-reported race/ethnicity, number of employees, number of years in business, WIC and SNAP (Supplemental Nutrition Assistance Program) participation, sells alcohol/tobacco, and store-related operational and structural characteristics. Differences in baseline characteristics by treatment group were compared using Fisher’s exact tests (for expected cell frequencies of <5) for dichotomous outcomes (i.e. WIC/SNAP participation, sells alcohol/tobacco, gender). Exploratory data analysis found that ANOVA assumptions of heteroscedasticity and non-normality were violated, therefore Kruskal–Wallis *H* tests were used to determine if there were any significant differences in continuous baseline characteristics (i.e. number of years in business, frequency of food deliveries, etc.) and outcomes for the intervention groups *υ*. control.

#### Impact analysis

To evaluate the effect of the interventions on storeowner psychosocial factors, and prices, stocking and sales of promoted foods, regression-based difference-in-difference models using linear generalized estimating equations with an independent correlation structure and robust se were used to account for within-subject correlation over time. Although we found no statistically significant differences in baseline characteristics according to intervention group, we suspect we had limited power to detect differences due to the relatively small sample size. For this reason, to test treatment effects, we employed difference-in-difference estimators to guard against baseline differences confounding the treatment effects. Working correlation structure was selected using the quasi-likelihood under independence model criterion (QIC)^(25)^. Three contrasts were tested: pricing only (G1) *υ*. control (G4); communications only (G2) *υ*. G4; and combined (G3) *υ*. G4. Outcome measures were analysed as dependent variables, with intervention group, time and a group × time interaction term as independent variables. The coefficient on the group × time variables is the ‘difference-indifference’ estimate, and its *P* value represents the test of whether the change in the outcome over time was statistically different from the change in the same outcome over time in the control group. The statistical software package Stata 13.1 was used for all analyses; statistical tests were two-sided with a significance level of *P* ≤0·05. One store (owner) allocated to the pricing only (G1) group dropped out during Phase 1 of the study due to health reasons and post-intervention data were not obtained. Therefore, impact data were analysed for twenty-three stores in total.

## Results

### Baseline characteristics and outcome variables

We found no statistically significant differences in baseline characteristics (Table 2) or baseline scores of outcome variables (Table 3) between the intervention groups and control.

### Change in healthy food availability (stocking of promoted foods)

Positive intervention effects were observed for the total stocking score for all promoted foods for all treatment groups *υ*. the control group. Pricing only, communications only and combined groups saw a 3·6 (95% CI 1·3, 5·9, *P* = 0·002), 2·5 (95% CI 0·7, 4·3, *P* = 0·007), and 3·5 (95% CI 0·8, 6·2, *P* = 0·012) unit score increase in stocking of promoted food types, respectively, compared with control (Table 3). Both pricing discount groups were associated with a larger effect than the communications only group, although *post hoc* tests did not find a statistically significant difference in magnitude between the three intervention types. When assessed by phase, intervention effects were significant only for Phase 1 drinks (G1: *β* = 0·8, 95% CI 0·2, 1·4, *P* = 0·01; G2: *β* = 1·3, 95% CI 0·6, 2·1, *P* = 0·001; G3: *β* = 1·3, 95% CI 0·1, 2·6, *P* = 0·03) and Phase 3 snacks (G1: *β* = 2·2, 95% CI 1·0, 3·4, *P* ≤ 0·001; G2: *β* = 1·8, 95% CI 0·6, 3·0) *P* = 0·003; G3: *β* = 1·3, 95% CI 0·5, 2·2, *P* = 0·002), but not for Phase 2 staple foods (G1: *β* = 0·7, 95% CI −0·6, 2·0, *P* = 0·3; G2: *β* = − 0·7, 95% CI −1·6, 0·3, *P* = 0·2; G3: *β* = 0·8, 95% CI −0·4, 2·1, *P* = 0·2).

### Changes in sales of promoted foods

No statistically significant changes in total promoted food sales were seen between the intervention groups and control (Table 3). There was a significant positive intervention effect of the combined pricing and communications intervention on Phase 3 snacks, observed as an increase of 6·4 units (95% CI 0·9, 11·9, *P* = 0·02) sold per day, *υ*. control.

### Changes in promoted food prices (pass-through)

Our secondary research question assessed whether the price discounts given to storeowners from the wholesaler were passed through to the customer. There was a significant treatment effect of the combined pricing and communications intervention on staple food prices (*β* = − 0·47, 95% CI −0·9, −0·03, *P* = 0·036), i.e. price decreased by $US 0·47 for all Phase 2 foods combined *υ*. control, but no effects were found for the other intervention groups. There were no other significant intervention effects on total promoted food prices compared with the control group.

### Change in storeowners’ psychosocial variables

In the pricing only, communications only and control groups, there were no statistically significant treatment effects on psychosocial factors between the intervention groups and control for all foods combined. However, intentions to sustain stock of Phase 3 snack foods increased in all intervention groups compared with control, trending towards significance (G1: *β* = 2·6, 95% CI −0·7, 5·8, *P* = 0·1; G2: *β* = 3·1, 95% CI −0·3, 6·5, *P* = 0·07; G3: *β* = 2·8, 95% CI −0·2, 5·7, *P* =0·06). Intentions to sustain stock of Phase 2 foods for pricing only (G1) and communications only (G2) groups actually decreased compared with control, trending towards significance (G1: *β* = −5·4, 95% CI −12·0, 1·2, *P* = 0·1; G2: *β* = − 4·4, 95% CI −9·5, 0·7, *P* = 0·09). There was a statistically significant decrease in outcome expectations for sales of Phase 1 drinks for G1 and G2 stores compared with control (G1: *β* = −3·4, 95% CI −4·9, −1·8, *P* = 0·001; G2: *β* = −2·6, 95% CI −5·2, −0·0, *P* = 0·05).

## Discussion

The present study is the first to evaluate the effect of store-directed price discounts on small store supply and sales of healthier foods, and the first to do so through wholesaler-supplied trade promotions. Additionally, the study addresses gaps in the literature that have called for factorial-designed intervention studies to show the interactive effects of price changes combined with additional non-price interventions^(26)^.

We found that all intervention groups (G1, G2 and G3) saw significant increases in stocking of promoted foods compared with control. Second, we found statistically significant increases in the sales of Phase 3 snack foods in the combined (G3) intervention group compared with control, and non-significant increases in G3 sales for all foods combined. No treatment effects were seen for sales in the pricing only (G1) or communications only (G2) group. Third, the increase in total sales was seen despite a lack of evidence of retail pass-through to customers in the combined (G3) group compared with control. Finally, there were no significant intervention effects on overall storeowner psychosocial factor scores compared with control, although treatment effects were found for phase-specific storeowner psychosocial factors.

Store-directed communications (e.g. small produce refrigerators, shelf talkers, posters, wholesale pamphlets) and store-directed price discounts (10–30 %) on promoted foods, separately and combined, encouraged increased stocking of healthier foods by storeowners. Combined pricing and communications intervention effects were not statistically different from intervention effects for either pricing only or communications only groups, showing that combined effects were not more than additive for promoted food stocking. The current study is consistent with other small store trials, which have reported increases in promoted food stocking through multiple approaches (i.e. coupons, structural change and health communications)^(6)^. The stock of promoted foods within small stores declined from baseline in the control group (while increasing in all intervention groups), demonstrating that simply ensuring the availability of healthier promoted foods at the participating wholesaler was insufficient to increase their purchase by storeowners.

When looking specifically at the different types of healthier options stocked in the three different intervention phases, statistically significant increases were seen in all intervention groups compared with control for Phase 1 beverages (as an increase of one type of healthier drink) and Phase 3 snacks (as an increase of two types of healthier snacks). Even modest increases in healthier food availability such as those shown herein can lead to healthier diets, as individual eating habits are largely determined by those food choices that are available^(4,27)^. No improvement was observed in the availability of Phase 2 healthy essentials (whole-wheat bread, canned tuna and frozen vegetables). We speculate several reasons for this. First, the wholesale price of a loaf of whole-wheat bread, after price discount, was $US 1 more than white or split-top wheat bread, a cost differential that likely deterred many storeowners from purchasing it. Second, storeowners would often confuse the two types of ‘wheat’ bread offered at the wholesaler (split top, 100% whole) and carry the less expensive split top in their stores during the intervention. With regard to frozen vegetables, only premium brand items (i.e. Hanover, Bird’s Eye) were included in the impact analysis. Process evaluation results (N Budd, unpublished results) indicated that those storeowners that stocked frozen vegetables chose to stock private-label brands because of their lower cost. Thus, if sales of private-label frozen vegetables increased in intervention groups compared with control, the SIQ would not have tracked this change.

The sales of promoted items also increased in the combined pricing and communications group (G3) compared with control, as an increase in total sales of promoted items that did not reach statistical significance at conventional levels and a statistically significant increase in sales of Phase 3 snacks. The increase in healthier snack sales was modest but suggests that PBA are both feasible and accepted by storeowners. The implications of choosing healthier snacks in this context is particularly important since high-fat and energy-dense snack foods are a common source of additional energy purchased by corner store customers in urban settings^(28–32)^. Given that the average American adult snacks two times or more per day^(33)^ and consumer packaged goods account for almost two-thirds of the energy consumed^(34)^, substitution of these foods with healthier snacks may provide the needed energy deficit for weight loss or weight maintenance. For example, replacing a 1506 kJ (360 kcal) honey bun (a common snack in this setting) with two low-fat granola bars (377 kJ (90 kcal) each) leads to a 753 kJ (180 kcal) deficit per snacking occasion. Furthermore, the demand for fresh, nutrient-dense, packaged snacks (i.e. hummus and pretzels, Greek yoghurt, baby carrots and dip) is increasing among convenience store customers nationwide^(35)^ and PBA in small urban food stores could provide a mechanism for healthier food suppliers and/or subsidiaries to gain footing in a setting replete with non-nutrient-dense snacks.

No statistically significant changes in sales were seen for the pricing only (G1) and communications only (G2) groups compared with control. Thus, while either the pricing or communications intervention alone motivated storeowners to stock, combined approaches may have been necessary to result in increased sales. A combined strategy would mimic the mechanism of an actual trade promotion, as food suppliers generally include storage and marketing materials to augment pricing incentives in order to support the sales of their promoted products (i.e. beverage coolers, point-of-sale displays, shelf talkers)^(36)^. Marketing research has found that trade promotions, even when pass-through does not occur, lead to an increase in sales^(14)^. Pertaining to the current study, it is possible that storeowners in the pricing groups felt some residual obligation to actively promote the foods themselves in return for receiving a wholesaler discount. Additionally, the pricing (G1) storeowners may have been more motivated to push the sales of promoted foods since their profit margins on the promoted items increased as a result of the wholesale discount (e.g. displaying promoted items in more prominent areas, etc.). The increase in promoted food profit margins coupled with in-store communications appeared to provide the combined (G3) storeowners with the necessary tools to increase healthier snack sales.

The two requirements of the store-directed PBA were to stock the promoted foods and provide retail-pass through to customers. The greatest changes in stocking of promoted foods were seen among the pricing groups (*n* 11), providing evidence that storeowners adhered, at least partially, to the first requirement of the PBA. For the most part, however, storeowners did not adhere to the second requirement (retail price discount pass-through), with the exception of Phase 2 foods ($US 0·47 difference in price changes for Phase 2 foods in combined stores *υ*. control). Storeowners may have provided retail pass-through for Phase 2 foods because of their perishability (i.e. bread, frozen vegetables) and high baseline cost compared with beverages and snacks.

We suggest several reasons why pricing intervention storeowners failed to consistently provide pass-through for the other foods. First, storeowners expressed concern over providing temporary price reductions because they believed they would result in customer complaints and distrust when prices were returned to normal levels^(37)^. Storeowners expressed that their customers were extremely price-sensitive, down to the smallest monetary unit^(37)^. Second, the limited research on trade promotions shows that 30% of trade promotions go directly to the retailer’s bottom line and this may have been the case with our stores^(14)^. Third, our staff had limited capacity to enforce the pass-through of the PBA because we did not have access to sales receipt data. Thus, there were no repercussions to the storeowner if pass-through did not occur, whereas in trade deals, the allowance is rescinded in the absence of the ‘performance’.

Finally, there were no significant intervention effects on overall storeowner psychosocial factor scores compared with control, although treatment effects were found for phase-specific storeowner psychosocial factors. There was a statistically significant decrease in outcome expectations for sales of Phase 1 drinks for pricing only (G1) and communications only (G2) stores compared with control. During the last month of the trial, a 5-cent-per-bottle tax was passed in Baltimore City, resulting in price increases of all bottled beverages at local wholesalers. Wholesale staff anecdotally commented that the bottle tax caused many storeowners to travel beyond the city limits to purchase food supplies (N Budd, unpublished results). Since intervention storeowners were obligated to purchase the promoted foods from the intervention wholesale stores, the 5-cent increase in price for each bottled beverage may have been enough of a price increase to cause a decrease in outcome expectations for these beverages, compared with control stores (who could shop at other sources beyond city limits).

There was a non-significant increase in storeowner intentions to sustain stocking of Phase 3 snacks in all intervention stores (G1, G2, G3) compared with control, which matches the significant increases in stocking (and sales for G3 stores) of these foods. Conversely, there was a non-significant decrease in intentions to sustain stock of Phase 2 staple foods among G1 and G2 storeowners compared with control, which is not surprising, considering that the stock and sales of these items did not increase during the intervention period.

### Limitations

There were limitations to the present study. First, we relied on storeowner recall to obtain sales data. Prior store-based trials have reported the complexity in obtaining sales receipt information from small independent stores and the current study was without exception^(22,38)^. To minimize the potential for reporting bias, pre-tested, standardized instruments were used^(22)^, and data collectors were extensively trained and standardized in their delivery. Second, the small produce refrigerators (or freezers) given to the twelve stores in the communications interventions were structural additions affecting healthier food supply and should be distinguished from the mechanisms of communications interventions which generally affect consumer demand. Third, post-intervention data collection was delayed substantially (i.e. ~3 months) following the trial’s end date, so that storeowners were not receiving any interventions at the time of collection. However, the delay likely muted intervention effects and provides evidence for sustainability at 3-month follow-up. Fourth, stocking and sales data on some promoted foods (i.e. fresh mixed fruit, grapes, cut melons, private-label brands of frozen vegetables) were excluded from the analyses because they were not collected at baseline. However, this likely led to more conservative results or an underestimation of intervention effects. Fifth, we were unable to collect data on the stocking and sales of unhealthier comparative foods (i.e. regular potato chips, cookies, regular sodas). Research on the effects of healthier food discounts on total energy purchased and consumed is mixed; but may lead to weight gain if substitution of healthier alternatives for unhealthy products does not occur^(39,40)^. Additionally, the generalizability of study results may be limited to low-income, urban, predominantly black neighbourhoods and stores. However, given the disproportionate burden of obesity and chronic disease placed on these subgroups, targeted interventions may be the most appropriate course of action. Finally, the PBA received by retailers in the present study are likely different from those offered by industry: discounts were offered on a few healthier items as opposed to a range of products; they were passed from the wholesaler to the retailer instead of directly from the manufacturer/supplier; and the communications strategy contained a ‘health education’ focus rather than a purely sales-driven one. The differences were partially due to fact that trade promotions have been used previously to push overall sales of suppliers’ products through a distribution channel, but they have not been used to drive the sales of only healthier foods or in small store settings. Using PBA in this context may require a different strategy and will require industry partnerships (e.g. a snack manufacturer) to bring applications to a larger and more realistic scale.

## Conclusions

The consumer packaged goods sector spends approximately $US 75 billion per year on trade promotions, compared with advertising expenditures of $US 37 billion^(14)^. Despite industry spending more on trade promotions than on any other marketing activity, academic researchers lack understanding about trade promotions, their effect on small, independent storeowners and the potential they hold to shift consumer preference^(12,14)^. Food access interventions must strive to create supportive environments for storeowners so that they feel confident they can stock and sell healthier food items without negatively impacting their bottom line. Scaled-up experimental research in real settings is needed to understand the mechanism by which trade promotions can increase healthy food supply and demand in small stores. Future efforts with stores should utilize scanner systems in order to examine own- and cross-price effects of trade promotions in these settings. Interventions should not focus solely on fruits and vegetables, but incorporate healthier alternatives to packaged snacks and beverages (i.e. chips, soft drinks), which are the most popular and profitable food items to small retailers in these settings (N Budd, unpublished results)^(41)^. Collaboration with beverage and snack food suppliers in these areas may reduce bottlenecks to healthier food access and enhance efficiency, as they have the infrastructure and materials to run trade promotions on their healthier product lines. Lastly, different types of trade promotion should be tested (i.e. slotting allowances, movement allowances, etc.) to determine which are most effective and feasible in small store settings. In a time of corporate self-regulation, incorporating trade promotions to increase healthy food access and demand has the potential to be a win–win for business owners’ bottom lines and public health alike.

## Figures and Tables

**Table 1:** B’More Healthy Retail Rewards (BHRR) intervention components and phases

			Communications examples (12 stores)					
				
Phase	Duration	Objectives	Interactive displays and taste tests	Educational handouts	Posters	Shelf labels and talkers	Giveaways	Promoted food discounts (12 stores)
1: Better Beverages	10 weeks; Feb–Apr 2013	Lower-calorie drink alternatives Replace soda with water Switch to low-fat milk	‘Rethink your drink!’	‘How does your drink measure up?’	‘Replace one bottle of XX with water each day to lose XX lbs per year!’	‘Refresh!’	Drink tumblers with BHRR logo	Deer Park Water – 25%
			Blind taste tests of popular drinks and lower-sugar/fat alternatives			‘Re-energize!’		Pepsi Next – 20%
						‘Refuel!’		Coke Zero – 20%
								Rutter’s 1% Milk – 20%
2: Healthier Essentials (Staple Foods)	8 weeks; Apr–Jun 2013	Replace white bread with whole-wheat bread Use frozen vegetables to increase vegetable intake Switch to tuna in water for a healthy lunch alternative	Banana/apple/whole-wheat bread pudding samples and recipes	‘What’s the difference between whole and refined grains?’	‘The Value of Frozen Vegetables’ (pick some up at your local corner store!)	‘Fiber-rich!’	Reusable cloth grocery bags with BHRR logo	Essential Everyday frozen vegetables (3 types) – 10%
						‘Wholey Delicious!’		Hanover & Bird’s Eye frozen vegetables (3 types) – 20%
			No-mayo tuna salad samples and recipes			‘Protein-Packed!’		100% whole-wheat bread – 20%
								Starkist & Bumblebee chunk light tuna – 10%
								Starkist & Bumblebee white albacore tuna – 20%
3: Healthier Snacks	8 weeks; Jun–Aug 2013	Replace sweets with lower-sugar/calorie alternatives Try baked potato chips instead of fried Have fresh fruit for a healthy snack	‘What’s in your snack?’	‘Easy and Quick Snacks 150 calories or less!’	‘Fresh Fruit Sold Here!’	‘Low-fat Snack Attack!’	Produce refrigerator or freezer with BHRR logo (store)	Utz Baked potato chips – 30%
			Baked chip taste test		‘Have a Snack Attack without the Fat!’			Quaker Oats 90 calorie granola bars – 15%
			Fruit salad samples			‘Baked is Better!’	Baked Chip Clips with BHRR logo (consumer)	Fresh fruit (apples, oranges, bananas) − 20%

**Table 2:** Baseline store and storeowner characteristics per treatment group; B’More Healthy Retail Rewards intervention conducted in twenty-four corner stores and two wholesale stores in Baltimore City, MD, USA, from February to August 2013

Store(owner) characteristic†,‡	Pricing only (G1; *n* 6)		Communications only (G2; *n* 6)		Combined pricing and communications (G3; *n* 6)		Control (G4; *n* 6)	
			
	*n*	%	*n*	%	*n*	%	*n*	%
Storeowner gender								
Male	6	100·0	5	83·3	5	83·3	6	100·0
Storeowner race								
Asian	5	83·3	4	66·7	4	66·7	4	66·7
African American	1	16·7	2	33·3	1	16·7	1	16·7
White	0	0·0	0	0·0	1	16·7	1	16·7
Storeowner ethnicity								
Hispanic/Latino	0	0·0	1	16·7	1	16·7	1	16·7
Storeowner is the primary food shopper for the store	5	83·3	5	83·3	4	66·7	6	100·0
WIC-approved	3	50·0	2	33·3	4	66·7	2	33·3
Accepts SNAP	6	100·0	6	100·0	5	83·3	5	83·3
Sells alcohol	1	16·7	2	33·3	0	0·0	0	0·0
Sells tobacco products	6	100·0	6	100·0	6	100·0	6	100·0
Checkout counter enclosed in Plexiglas	6	100·0	6	100·0	4	66·7	5	83·3
Behind-the-glass§	2	33·3	2	33·3	1	16·7	1	16·7
Store(owner) characteristic‖	Mean	sd	Mean	sd	Mean	sd	Mean	sd

Number of times storeowner shopped for his/her store in the past 30 d	33·6	15·5	43·9	13·2	44·0	21·8	38·7	6·6
Number of times storeowner shopped at participating wholesale stores in the past 30 d	17·8	9·4	25·0	8·4	15·8	12·8	21·0	9·1
Number of employees (incl. family members. excluding owner)	2·5	1·8	2·2	0·8	3·8	2·3	2·3	1·9
Number of years in business	11·1	9·9	8·1	5·1	4·2	2·9	14·5	8·4
Average number of unique customers per day	147·0	85·0	154·0	103·0	145·5	145·0	196·0	87·0
Number of beverage refrigerators	2·8	0·8	4·3	2·9	3·3	1·4	3·7	1·4
Number of additional refrigerators (incl. deli cases)	1·5	1·2	1·2	0·4	1·0	1·1	1·3	0·5
Number of freezers	3·0	1·1	2·7	1·0	2·8	0·4	3·1	0·8
Frequency of food/beverage deliveries in the past 30 d	16·7	10·5	10·7	8·8	15·7	12·7	21·5	11·3

WIC, Special Supplemental Nutrition Program for Women, Infants, and Children; SNAP, Supplemental Nutrition Assistance Program.

† No significant differences found between treatment groups (*P* > 0·05).

‡ Fisher’s exact test (for expected frequencies of <5) for dichotomous outcomes.

§ ‘Behind-the-glass’ stores are characterized by having barriers of Plexiglas walls separating the consumer on one side from the retail items and store employees on the other side.

‖ Means reported using one-way ANOVA for continuous outcomes (for interpretation). Differences between groups determined using Kruskal–Wallis tests.

**Table 3:** Treatment effects for intervention groups compared with control; B’More Healthy Retail Rewards intervention conducted in twenty-four corner stores and two wholesale stores in Baltimore City, MD, USA, from February to August 2013

	Pricing only (G1; *n* 5)				Communications only (G2; *n* 6)				Combined pricing and communications (G3; *n* 6)				Control (G4; *n* 6)		
				
Measure	Baseline score	se	Change from baseline	Diff. in diff.†	Baseline score	se	Change from baseline	Diff. in diff.†	Baseline score	se	Change from baseline	Diff. in diff.†	Baseline score	se	Change from baseline
Stocking score															
Phase 1 beverages‡	1·2	0·4	0·4	0·8*	1·2	0·8	1·0	1·3***	1·7	0·8	1·0	1·3*	1·5	0·5	−0·3
Phase 2 staple foods	1·7	1·0	0·5	0·7	1·5	1·0	−0·8	−0·7	1·8	1·2	1·0	0·8	1·5	0·5	−0·2
Phase 3 snack foods	1·3	1·5	1·7	2·2***	1·0	1·1	1·3	1·8**	2·2	1·5	0·8	1·3**	1·2	0·8	−0·5
All foods combined	4·2	2·3	2·6	3·6**	3·7	1·6	1·5	2·5**	5·7	2·3	2·5	3·5*	4·2	1·6	−1·0
Sales (units)															
Phase 1 beverages	15·7	12·9	5·1	3·3	22·7	29·3	−8·9	−10·7	10·0	5·0	7·7	5·9	14·6	10·3	1·8
Phase 2 staple foods	8·3	12·1	−6·1	−5·6	1·4	2·1	−0·9	−0·3	4·3	4·2	−1·1	−0·6	2·5	2·9	−0·6
Phase 3 snack foods	10·6	18·8	−0·9	3·6	5·7	8·0	−1·5	2·9	12·9	13·9	2·0	6·4*	9·6	8·7	−4·4
All foods combined	34·5	31·8	−2·0	1·2	29·8	29·2	−11·3	−8·1	27·2	18·3	8·6	11·8	26·6	13·1	−3·2
Promoted food prices§ ($US)															
Phase 1 beverages‖	7·14	2·22	0·00	0·00	7·76	5·38	−0·55	−0·55	8·30	5·41	0·16	0·16	4·18	3·54	0·00
Phase 2 staple foods	7·64	3·33	0·16	0·09	4·27	2·53	0·02	−0·04	7·77	5·48	−0·40	−0·47*	4·56	3·06	0·07
Phase 3 snack foods	1·76	0·82	0·15	0·14	1·23	1·16	0·17	0·16	2·46	1·61	0·02	0·01	1·63	2·21	0·01
All foods combined	16·55	2·12	0·31	0·24	13·26	8·57	−0·35	−0·43	18·52	11·72	−0·22	−0·30	10·38	6·50	0·08

* *P*≤0·05,

** *P*≤0·01,

*** *P*≤0·001.

† Unless otherwise noted, treatment effect estimates were derived from difference-in-difference analyses using linear generalized estimating equations with independent correlation structure and robust se (change in intervention scores from baseline – change in control scores from baseline).

‡ Exchangeable correlation structure used.

§ Baseline score indicates the pooled prices of foods per phase of those foods that were stocked. If a food was not stocked at either time point, the price was given a value of 0 for both pre and post measurements, so total change was 0 for these foods.

‖ Unstructured correlation structure used.
